# Highly Upregulated Lhx2 in the Foxn1^−/−^ Nude Mouse Phenotype Reflects a Dysregulated and Expanded Epidermal Stem Cell Niche

**DOI:** 10.1371/journal.pone.0064223

**Published:** 2013-05-16

**Authors:** Stefan Bohr, Suraj J. Patel, Radovan Vasko, Keyue Shen, Guofeng Huang, Martin L. Yarmush, Francois Berthiaume

**Affiliations:** 1 Center for Engineering in Medicine, Shriners Hospitals for Children and Department of Surgery, Massachusetts General Hospital, Harvard Medical School, Boston, Massachusetts, United States of America; 2 Department of Medicine, New York Medical College, Valhalla, New York, United States of America; 3 Department of Biomedical Engineering, Rutgers University, New Brunswick, New Jersey, United States of America; University of Birmingham, United Kingdom

## Abstract

Hair cycling is a prime example of stem cell dependent tissue regeneration and replenishment, and its regulatory mechanisms remain poorly understood. In the present study, we evaluated the effect of a blockage in terminal keratinocytic lineage differentiation in the Foxn1^−/−^ nude phenotype on the epithelial progeny. Most notably we found a constitutive upregulation of LIM homeobox protein 2 (Lhx2), a marker gene of epithelial stem cellness indispensible for hair cycle progression. However, histological evidence along with an erratic, acyclic rise of otherwise suppressed CyclinD1 levels along with several key markers of keratinocyte lineage differentiation indicate a frustrated expansion of epithelial stem cell niches in skin. In addition, CD49f/CD34/CD200–based profiling demonstrated highly significant shifts in subpopulations of epithelial progeny. Intriguingly this appeared to include the expansion of Oct4+ stem cells in dermal fractions of skin isolates in the Foxn1 knock-out opposed to wild type. Overall our findings indicate that the Foxn1^−/−^ phenotype has a strong impact on epithelial progeny and thus offers a promising model to study maintenance and regulation of stem cell niches within skin not feasible in other in vitro or in vivo models.

## Introduction

**Figure 1:**
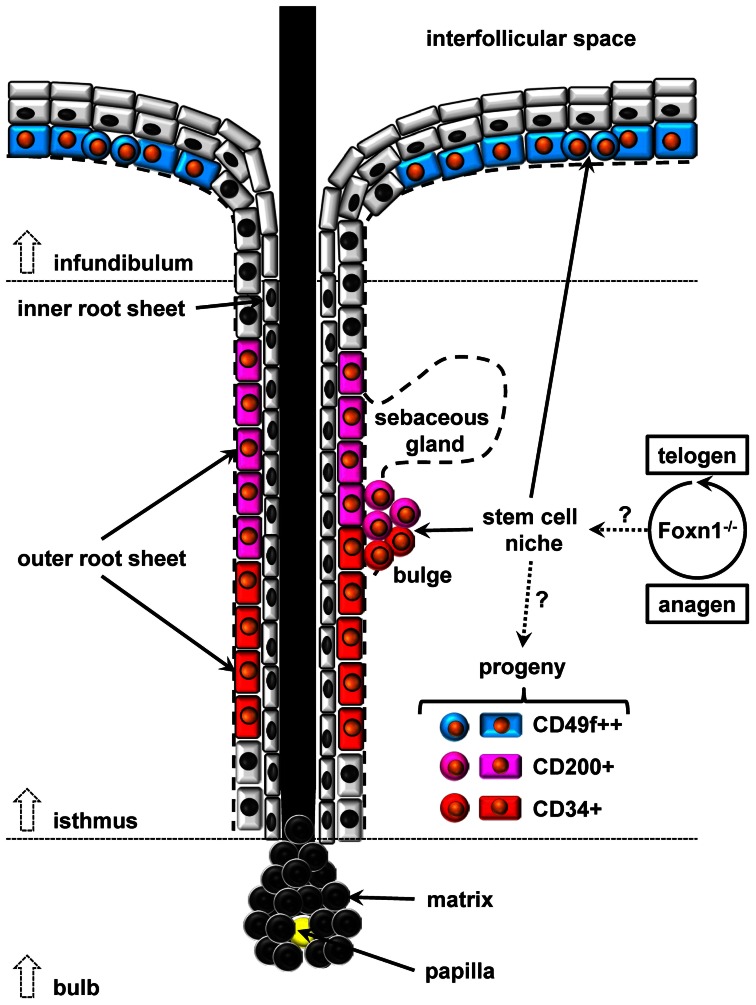
**Schematic of mature, anagen hair follicle (HF) morphology with typical localization of keratinocytic lineage progeny in wild type (WT).** CD49f/α6-integrin (blue) positive epithelium dominates within the basal layer of the surface epithelium. CD200 (purple) and CD34 (red) are both strongly expressed within the bulge region. In addition, CD200 is also expressed in upper parts of the outer root sheet (ORS) whereas CD34 expression may extend below the bulge region. Matrix cells of the hair bulb are considered CD49f/CD200/CD34 negative. In general, surface markers of epithelial progeny are not exclusive for each other in part depending on the stage of hair cycling. This study evaluates various effects of Foxn1 loss-of-function on telogen-anagen hair cycling and epithelial stem cell niche regulation in Nu/Nu mice.

Skin fosters its own multipotent epithelial progeny in order to ensure keratinocytic lineage replenishment necessary for maintaining barrier function, a cyclic anagen-catagen-telogen sequence of hair growth [1], and wound healing [2], [3], [4]. At least two distinct epidermal stem cell niches have been described. The first niche is situated in the basal layer of the interfollicular space and the other one in the bulge region [5] of the hair follicle (HF), an area located shortly before the isthmus widens into the infundibular space and which is typically found in close proximity to the sebaceous glands of HF’s [6], [7], [8]. In this context, we studied several proposed markers of progeny with localized expression within the epithelium and the hair follicles (Fig. 1). More specifically, we investigated the impact of Foxn1 loss-of-function on epithelial progeny in order to establish the nude mouse (Nu/Nu) phenotype as a valuable model to study mechanisms of stem cell regulation in skin.

Knock-out (KO) of the Foxn1 (Forkhead box protein N1) gene results in two well-known, yet independent, defects in mice: hairlessness and athymia. The latter is linked to a Foxn1 dependent differentiation of thymic epithelial cells which in turn are crucial for T-cell selection and maturation [9], resulting in a severe, partial T-cell immune deficiency and tolerance. In contrast, a current understanding of the ‘hairless’ phenotype is limited. In embryonic and fetal stages, dermal organogenesis of these animals, including HF formation, appears to be undisturbed. Following birth, the commencement of hair follicle growth initially appears normal, but in further postnatal development exhibits severe morphological disturbances with failure of newly formed hair shafts to penetrate the overlying epidermal surface. Eventually, an abrogation of hair shaft formation occurs [10]. Thus, in Nu/Nu mice normal hair cycling is severely disturbed and a distinction of telogen vs. anagen ambiguous. Our histological evaluation confirmed that skin on the dorsum of Nu/Nu mice is truly hairless with no sign of active hair formation most of time. However, spontaneous formation of rudimentary hair shafts/lanugo hair occurs throughout adulthood, mimicking some aspects of the anagen. Target gene analysis has revealed Foxn1 as a crucial transcriptional regular of several hair keratin genes, both in rodents and humans [11]. Moreover, induction of Foxn1 expression resulted in growth arrest and differentiation of stem cell founder clone colonies in vitro [12]. As shown by Calautti et al., phosphoinositide 3 (PI 3)-kinase signaling to Akt-regulated transcription plays a crucial role in Foxn1^−/−^ induced differentiation of keratinocytes [13].

We hypothesized that a functional blockage in the terminal differentiation of keratinocytes present in Nu/Nu mice would affect the regulatory mechanisms which control stem cell niches in skin. In this respect, we herein demonstrate that different subpopulations of epithelial progeny as well as several regulatory pathways differ significantly from the wild type (WT) condition. Most notably we found the transcription factor Lhx2, a putative regulator of hair cycling and stem cell regulation, strongly and constitutively induced in Nu/Nu mice, as opposed to WT.

## Methods

### Experimental Mice

C57BL/6 wild type (WT) and Foxn1^−/−^ (B6.Cg-Foxn1nu/J) nude mouse strain were obtained from The Jackson Laboratory (Bar Harbor, ME, USA). Ethics Statement: Animals were housed under standard conditions in an institutional animal facility in accordance with *National Research Council* guidelines and procedures performed on animals for this study were approved by the *Subcommittee on Research Animal Care* at Massachusetts General Hospital, Boston, MA. To induce hair growth in WT mice, animals were anesthetized by intraperitoneal injection of ketamine/xylazine followed by clipping and uniform removal of hair roots using hair wax and depilation cream (NAIR™). Primary epithelial lineage cells were isolated from newborn skin and used in FACS analyses and cell culture experiments.

### Imaging & Histology

Close-up images of skin were taken using a macroscope (Olympus SZX12) mounted with a digital camera (Olympus DP10, JPN). Histological evaluation of hair growth was performed on 10 µm sections of formalin fixed and paraffin embedded dorsal skin samples followed by hematoxylin and eosin (H&E) or immunohistology (IHC) using standard protocols (//www.ihcworld.com/). IHC, including negative controls, for Lhx2 was performed using the following antibodies and detection reagents: goat anti-Lhx2 (//us.acris-antibodies.com; #AP16774PU-N), rabbit anti-goat (//www.abcam.com; #ab6740), Vectastain ABC Kit™/DAB Peroxidase Substrate Kit™ and methyl green nuclear counterstain (all//www.vectorlabs.com; #PK-4000, # SK-4100, #H-3402). Images were taken using a Nikon Eclipse E600™ microscope (//www.nikoninstruments.com) mounted with a Spot PCI-CE™ camera (//www.spotimaging.com).

### Cell Isolations

Keratinocytes were isolated from skin of newborn animals up to three days post birth (NBD1-3). Euthanized animals were sterilized by immersion in 90% ethanol for 5 min followed by sterile processing. Dissected skin was floated overnight at 4°C on trypsin (www.invitrogen.com)-buffer followed by separation of epidermis from dermis as previously described [14]. For the isolation of a hair follicle fraction, separated dermis was cut in small pieces and partly digested with 0.3% type IV collagenase in Williams E medium (both Sigma Aldrich, St. Luis, MO, USA)//www.sigmaaldrich.com) for additional 20 min at 37°C followed by vigorous vortexing. Epidermal fractions were filtered through 40 µm, dermal fractions through 100 µm strainers. In FACS analysis, a further digesting step in 0.1% trypsin in WilliamsE for 20 min at 37°C and 40 µm straining prior to cell labeling was performed.

### FACS

Epithelial isolates from newborn skin were labeled in 1x PBS buffer supplemented with 0.5% fetal calf serum (both www.invitrogen.com) at 4°C for 40 min using following antibodies with isotype controls: APC-CD49f/α6-integrin (//us.ebioscience.com; #17-0495-80; isotype: #17-4321), PE-CD34 (//www.bdbiosciences.com; #551387; isotype: #551799), PerCP-CD200 (//us.ebioscience.com; #46-5200-80; isotype: #45-4321 ). Labeling with PE-Oct3/4 (//us.ebioscience.com, #12-5841-80; isotype: #551799) required cell permeabilization using BD Cytofix/Cytoperm™ Plus (//www.bdbiosciences.com; #555028). Sorting of labeled isolates were performed using either a BD FACSCalibur™ with CellQuestPro™ or a BD SORP FACSAria IIs™ platform with BD Diva™ operating software (//www.bdbiosciences.com) and data was further analyzed using FlowJo™ software (//www.flowjo.com).

### Epithelial Cell Culture

Fresh epithelial isolates were cultured as previously described [14], [15], [16]. Briefly, to allow initial attachment, cells were incubated (<8h) in 0.2 mM/intermediate calcium media (ICM) [14], [15], [16] on collagen I (∼0.125 mg/ml) and laminin (∼0.25 mg/ml) (both//www.invitrogen.com; #A1048301, #23017015) coated dishes. Media were then changed to low calcium conditions using: (1) low calcium media (LCM) with serum [14], [15], [16] or (2) serum-free KGM-Gold™ keratinocyte growth media (//www.lonza.com; #192060). Seeding density for isolates on a 10 mm dish was the equivalent of roughly one newborn animal or 1–1.5 M cells. Cells were cultured at 34°C and 5% CO_2_ for three days before evaluation.

### Gene Expression Analysis

RNA was extracted from cells or tissue samples using either *TRIzol® Reagent* extraction (//www.invitrogen.com) or *AllPrep DNA/RNA/Protein Mini Kit®* (//www.qiagen.com). Total RNA quality was assessed by spectroscopy (NanoDrop®ND-1000,//www.thermoscientific.com) and micro fluidic gel analysis (Agilent® RNA 6000 Nano Chip/Agilent®2100 Bioanalyzer;//www.agilent.com) followed by cDNA conversion using the *SuperScript™III Platinum®Two*-*Step qRT-PCR Kit* (//www.invitrogen.com) or a *RT^2^ First Stand Kit® C-03* (//www.sabiosciences.com) and a *Perkin Etus Thermal Cycler 480* (www.perkinelmer.com). The gene expression patterns were assessed by quantitative PCR (qPCR) with a *RT^2^ SYBR® Green/Rox qPCR Master Mix* (//www.sabiosciences.com) using a *Stratagene™ mx3005P* system (//www.genomics.agilent.com). A melting curve was used to confirm the specificity of each primer pair. Each sample was run in triplicate to exclude outliers. Gene expression was analyzed using the ΔΔCT-method (*RT^2^qPCR Primer Assay User Manual, Version2.17,/*/www.sabiosciences.com) with GAPDH as the normalizing gene. GAPDH primer was purchased (//www.sabiosciences.com). The following primers were designed using *Primer3 v. 0.4.0* software (//frodo.wi.mit.edu/primer3) utilizing DNA coding sequences (//www.ncbi.nlm.nih.gov/nuccore): Cateninβ, forward 5′-GTCCGAGCTGCCATGTTC-3′, reverse 5′-CAAGTTCCGCGTCATCCT-3′; CD200/Ox2, forward 5′-TGCTCCTTGCTGCGATTCC-3′, reverse 5′-TGGCTTGCTCGCTGCAGTA-3′; CD200R1, forward 5′-TGGGCAGGAACATCACCT-3′, reverse 5′-CTGCCATTGCCTCACAGA-3′; CyclinD1, forward 5′-GCGTACCCTGACACCAATCT-3′, reverse 5′-CACAACTTCTCGGCAGTCAA-3′; DeltaL4, forward 5′-GTGGGGGATACATTCATTGC-3′, reverse 5′-TCAGCCAAATCATCCAA-3′; Foxn1, forward 5′-CGAAGGTCACCAGCCACT-3′, reverse 5′-CCAGCCATCAGGAGCAGT-3′; Fzd5, forward 5′-ACACTCCCAGGACCACCA-3′, reverse 5′-AGACCACAGGCCAATCCA-3′; Gli1, forward 5′-TTCGATTCCCAGGAGCAG-3′, reverse 5′-CTTCTCGCCCGTGTGTCT-3′; Gli2, forward 5′-GCACGTTCGAAGGCTGTT-3′, reverse 5′-GAGTGCGGTTCTGGTGCT-3′; Gli3, forward 5′-GCGAGTCTGCAGTGAGCA-3′, reverse 5′-TCTCTGGTGCAGCCTTCC-3′; Krt14, forward 5′-AGGGTGCTGGATGAGCTG-3′, reverse 5′-CAGCATCCTTGCGGTTCT-3′; Krt15, forward 5′-GGCAGAGATGAGGGAGCA-3′, reverse 5′-CATAGCGGCACTCCACCT-3′; Lhx2, forward 5′-TGGCAGTAGACAAGCAATGG-3′, reverse 5′-TGTGCATGTGAAGCAGTTGA-3′; Oct4, forward 5′-CGGAGGGATGGCATACTG-3′, reverse 5′-CCTTCTGCAGGGCTTTCA-3′; Ptch1, forward 5′-CGGTGTCTGGCATCAGTG-3′, reverse 5′-GGGTCAAGGGAGGCTGAT-3′; Sox2, forward 5′-AGCGCCCTGCAGTACAAC-3′, reverse 5′-TGATCATGTCCCGGAGGT-3′; Shh, forward 5′-GCAGACCGGCTGATGACT-3′, reverse 5′-CGTGGTGATGTCCACTGC-3′; Smo, forward 5′-TGCTGCTGCTGGTACTGC-3′, reverse 5′-CACGTTGTAGCGCAAAGG-3′; Wnt5a, forward 5′- GAAGCAGGCCGTAGGACA -3′, reverse 5′-CGCCGCGCTATCATACTT-3′; Wnt10b, forward 5′-AACTGCTCGGCACTGGAG-3′, reverse 5′-CCGGTCTTGCTCACCACT-3′.

### CyclinD1 as a Marker of Hair Cycling

Using qPCR, the dynamic change of CyclinD1 mRNA expression levels from whole skin where measured over time following depilation/induction of hair growth in wild type animals. The telogen or anagen state of skin samples used for mRNA quantification was confirmed by histology. As previously described, CyclinD1 levels during hair cycling mostly reflect mitogenic activity in the bulge region and the outer root sheet of HF’s but not of matrix cells in the bulb region of HF’s [17]. Skin samples with a telogen appearance in histology consistently showed lowest CyclinD1 mRNA levels and where used as a reference to determine the state of hair cycling in the Nu/Nu phenotype.

### Statistics

The results are presented as mean±SD and/or SEM. Sample size refers to the number of tissue samples (equal to the number of mice) analyzed. Pair wise comparisons were performed using a 2-tailed Student t test. These analyses were performed using Prism®4.0 (//www.graphpad.com/) software. A 2-tailed P<0.05 was considered statistically significant.

## Results

### CyclinD1 Expression Levels as a Putative Hair Cycle Marker in Nu/Nu Mice

**Figure 2:**
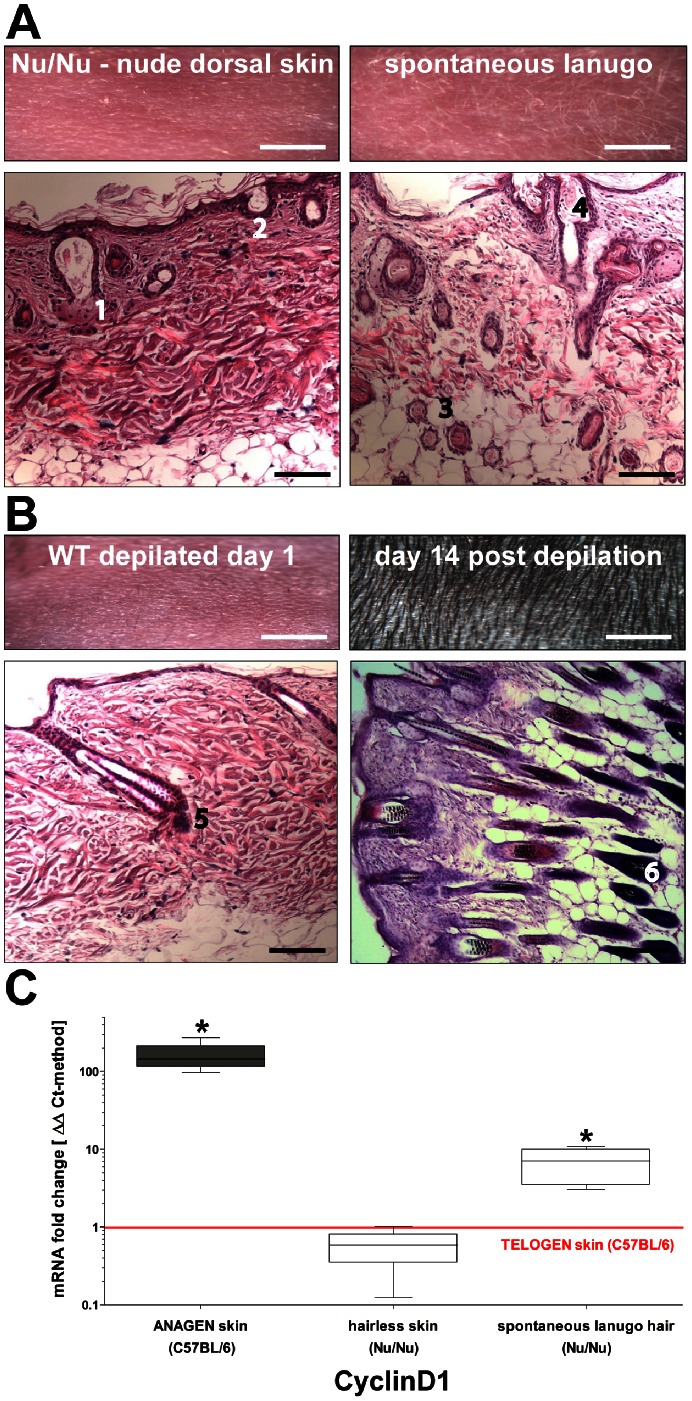
**Morphological features along with CyclinD1 levels suggest a continuous, yet frustrated activation of hair cycling within the nude mouse phenotype.** (A) The Foxn1^−/−^ (Nu/Nu) phenotype demonstrates spontaneous lanugo hair growth as opposed to a complete hairless condition. Histologically, superficial epithelial cysts/pseudo-infundibula which often fail to connect to the surface and associate with sebaceous gland cells (1) represent the equivalent of HF’s in wild type (WT). In addition, invaginations of a hyperplastic epithelium (2) resemble stages of de novo embryonic HF formation. With spontaneous lanugo hair formation, a dysmorphic root sheet (RS) (3) and infundibulum (4) containing a rudimentary hair shaft can be seen. (B) In WT telogen, a dermal papilla complex (5) gets activated by depilation and a newly forming RS expands both in width and length into the deeper dermis (6) in order to generate several terminal hair shafts. (C) Comparative CyclinD1 mRNA levels are suggestive for a telogen and anagen equivalent in the Nu/Nu phenotype (qPCR/ΔΔCt-method: n = 6; *P<0.05). The red horizontal line represents the telogen skin level used as reference. Scale bars: 4 mm (macroscopic); 50 µm (microscopic).

In contrast to WT mice, the rhythmic change between active hair growth (anagen) and a non-proliferative resting phase (telogen) is less obvious in the Nu/Nu phenotype. The study by Xu et al. revealed that CyclinD1 reflects stem cell proliferation in the bulge area and suggests it is a key marker of mitogenic activity within this area of hair follicles during cycling (HF) [17]. Therefore, we compared the dynamic change of CyclinD1 mRNA expression levels during hair cycling in WT animals with Nu/Nu phenotype (also see methods). Macroscopically, we saw that Nu/Nu mice spontaneously developed patches of skin with lanugo hair (as opposed to terminal hair shaft growth), resembling the typical wave pattern of hair growth in WT mice (Fig. 2A). Detailed histological examination confirmed only rudimentary HF’s located superficially in otherwise completely hairless skin. With spontaneous lanugo hair formation, hair follicles were able to expand into deeper dermal layers and formed several features that resembled terminal hair shaft morphology in WT mice (Fig. 2A, B). CyclinD1 expression in samples of Nu/Nu mice was correlated to the telogen and anagen states in WT mice as defined by histology and CyclinD1 mRNA levels. We showed that spontaneous lanugo in Nu/Nu mice was accompanied by an almost 10-fold increase in CyclinD1 levels. Nevertheless, irrespective of this robust up-regulation of CyclinD1, the absolute levels remained significantly lower (ten times) compared with the anagen state in WT mice. However, completely hairless Nu/Nu skin exhibited lower CyclinD1 expression levels compared to the WT mice telogen state (Fig. 2C). These data demonstrate that despite strongly suppressed mitogenic activity within the bulge stem cell niches in Nu/Nu mice, hair cycling between the telogen and anagen states remains functional.

### The Embryonic Stem Cell Markers Sox2 and Oct4 are Induced During Early Anagen in WT but are Constitutively Suppressed in Nu/Nu Mice

**Figure 3:**
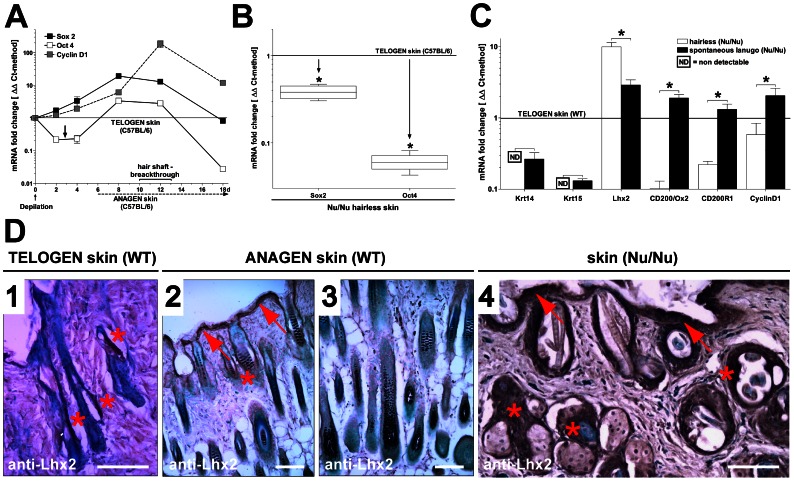
**Comparative expression of stem cell markers in the Nu/Nu phenotype vs. WT.** (A) Following induction of a new anagen hair cycle by depilation, WT skin shows a distinct dynamic expression of the embryonic stem cell (SC) markers Sox2 and Oct4. (B) Conversely, in the Nu/Nu phenotype, both markers are permanently suppressed (qPCR/ΔΔCt-method: n = 4; *P<0.05). (C) Other epithelial lineage SC markers show distinct changes in expression compared to WT, notably of Lhx2. (D) IHC confirms a very localized Lhx2 expression in the bulge region (asterisk) of WT telogen (D1) HF’s that extents to the infundibulum and the epithelial surface (arrow) during anagen (D2/3). In the Nu/Nu phenotype Lhx2 is strongly expressed in both hyperplastic surface epithelium and bulge region proximity. Scale bars: 100 µm (D).

HF’s of adult individuals in rodents, in contrast to human HF’s, are most of the time in a telogen state and following depilation, WT mice usually regrow fur within 3 weeks and reenter the telogen state afterwards. A switch from telogen to anagen requires a normally quiescent stem cell population to enter a proliferative phase in order to give rise to more committed lineage progenitors, which in turn will sustain the anagen growing phase. To scrutinize this assumed change in stem cell behavior, we induced a new anagen hair cycle in WT mice (via depilation) and examined mRNA expression of stem cell marker genes SRY-sex determining region Y-box 2 (Sox2) and octamer-binding transcription factor 4 (Oct4/POU5F1) over time together with changes in CyclinD1 mRNA levels. Both, Sox2 and Oct4 are transcription factors supposed to be critical for self-renewal of undifferentiated embryonic stem cells, and their activation has been used to generate adult pluripotent stem cells from various tissues including skin epithelium [18]. In WT mice, we observed a continuous increment of Sox2 levels of more than 20-times higher than in telogen control, which was followed by a steady decline under sustained high proliferative activity/active hair growth. Interestingly, Oct4 expression showed a transient but significant decrease following depilation of animals that was followed by a 2-fold increase, similar to the expression pattern of Sox2 (Fig. 3A). Hence, Sox2 and Oct4 levels were significantly elevated during the early, but not late stages of anagen.

Next, we compared Sox2/Oct4 expression levels in hairless Nu/Nu mouse skin (assumed telogen) to the WT telogen and saw a strong suppression of both stem cell markers, despite similar CyclinD1 levels in all samples (Fig. 3B).

### As Opposed to other Stem Cell Markers, Lhx2 is Constitutively Up-regulated in the Nu/Nu Phenotype

We proceeded to characterize expression levels of recognized bulge stem cell markers in proportion to WT telogen. Keratin 14 and 15 (Krt14&15) mRNA was not detectable in hairless- and strongly suppressed in lanugo-Nu/Nu skin. Similarly, the cell surface glycoprotein CD200/Ox2 and its receptor CD200-R1 [19], [20] were substantially reduced in hairless- but reached basal WT telogen levels in lanugo-Nu/Nu skin. Most significantly, the expression of Lhx2 was increased almost 10-fold in hairless-Nu/Nu skin and remained upregulated with spontaneous lanugo hair formation. Furthermore, suppression of stem cell markers in hairless-Nu/Nu skin (compared to WT telogen) all in all resembled the observed suppression in the later stages of WT anagen (day 12 of depilation), with the exception of Lhx2 (Fig. 3C).

To specifically localize and confirm Lhx2 expression at the translational level, we performed anti-Lhx2 immunohistochemistry (IHC) on representative skin tissue sections. In WT telogen HF’s, Lhx2 positivity could almost exclusively be detected in the stem cell-containing bulge region. Conversely, in late WT anagen with highly proliferative terminal hair growth, Lhx2 positive cells were predominately localized in the surface epithelium with expression in bulge region being less evident. In contrast to WT mice, IHC demonstrated strong expression of Lhx2 within the bulge region and interfollicular epithelium of Nu/Nu animals, along with a highly hyperplasic appearance with an increase in ‘basal cell-like’ epithelial layers compared to WT (Fig. 3D).

### Defining Epithelial Progenitor Cell Populations in Nu/Nu vs. WT Newborn Skin

**Figure 4:**
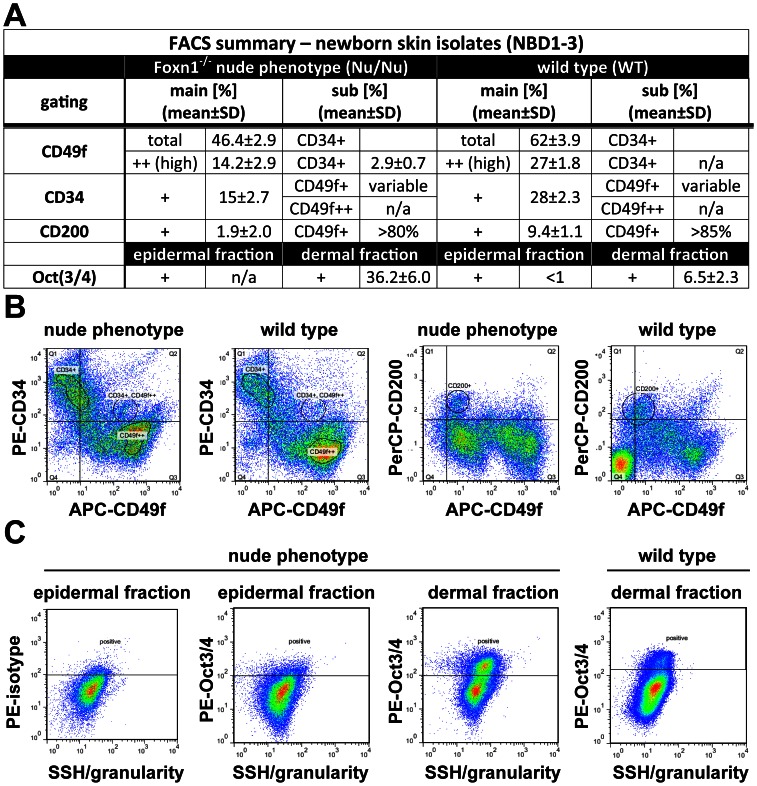
**As Oct3/4+ population is expanded; CD49f+, CD34+ and CD200+ populations are reduced in epithelial isolates from newborn skin of the Foxn1^−/−^ phenotype vs. wild type.** (A) Flow cytometry summary table listing fractions positive for markers of progeny within epithelial isolates from newborns (%±SD, n>6). Distinct differences between the Foxn1^−/−^ phenotype and wild type are demonstrated by comparative sample flow cytometry data gated for (B) CD49f, CD34, CD200 and (C) Oct3/4 positive epithelial fractions.

We further evaluated impact of Foxn1 loss-of-function in the Nu/Nu phenotype on progenitor cell fractions compared to WT mice using flow cytometry (see detailed summary in table Fig. 4A). The following markers of epithelial progeny were evaluated in epithelial isolates from newborn skin up to day 3 post birth (NBD1-3): CD49f (α6-intergrin) [21], [22], CD34 [23], CD200 (syn.: Ox2) [24], [25], [26] and Oct(3\4) [27].

In total (epidermal+dermal fraction) epithelial isolates, we saw distinct differences between the progenitor populations. The most widely spread CD49f positive population in WT-NBD1’s (>58%) was significantly reduced in Nu/Nu-NBD1’s (<50%) with a high positive CD49f (++) population averaging above 25% in WT and below 18% in Nu/Nu animals. Similarly, a CD34 positive population that can be predominantly localized below the isthmus level of HF’s appeared to be reduced in the Nu/Nu phenotype (>18%) compared to WT (>25%) (Fig. 4B). Finally, we detected CD200, an established marker of epithelial progenitors situated in the bulge region most negatively affected in Nu/Nu-NBD1 (<4%) skin with a 50% reduction compared to WT-NBD1 (>8%) skin (Fig. 3B). Selective gating (in order to identify double or triple positive subpopulations in general) showed that populations that were highly positive for CD49f or CD34 were exclusive to each other in WT. However, a highly variable ‘grey zone’ of CD49f+/CD34+ populations exists. Remarkably, we were able to identify a distinct CD49f++/CD34+ (>2%) population in Nu/Nu-NBD1 isolates which appears to be only marginally present in WT (Fig. 4B). Similarly, CD200 and CD34 were mutually exclusive as well but a CD200+ population was typically identified within CD49f+, but not CD49f++ population.

Using a cell-permeabilizing labeling technique, we also evaluated intracellular expression of Oct(3\4), a more general stem cell marker, in NBD1 epidermal and dermal fraction isolates (Fig. 4C). In Nu/Nu-NBD1 skin, we saw a large Oct(3\4)+ (>30%) population in the dermal fraction which was virtually absent within epidermal fractions. In comparison, WT-NBD1 dermal fractions contained a much smaller Oct(3\4)+ (<9%) population that also appeared within epidermal isolates. Our findings indicate that with the induction of a first anagen hair cycle post birth as well as in adult skin, a multipotent epithelial progenitor population is periodically expanded as the consequence of Foxn1 loss-of-function. The lack of Oct(3\4)+ population in epidermal fractions from Nu/Nu mice, as opposed to WT-NBD1 skin, is probably a consequence of a failure of HF’s to connect to the surface (Fig. 3D-4), resulting in a distinct separation of HF compounds from surface epithelium during isolations.

Overall, the Foxn1^−/−^ phenotype is highly informative in demonstrating the effects of a blockage in the expression of keratins on epithelial lineage differentiation, even down to the level of the least-committed epithelial stem cells.

### Wnt-signaling in the Nu/Nu Phenotype Varies Considerably from WT

**Figure 5:**
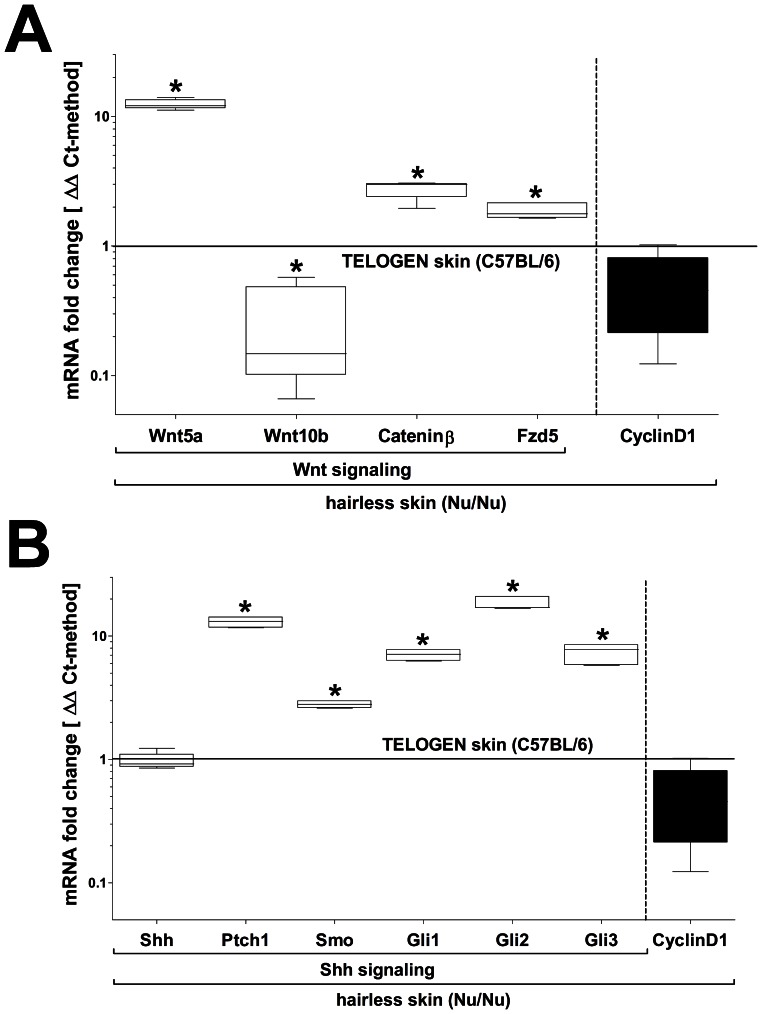
**Alterations in Wnt- and Shh-signaling component expression in the Nu/Nu phenotype.** (A) The canonical (Wnt5a/Cateninβ increased) and non-canonical (Wnt10b suppressed) Wnt-pathway is conversely altered in the Nu/Nu phenotype. (B) Also, with the exception of Shh itself, downstream components of Shh-signaling are upregulated in the Nu/Nu phenotype compared to WT telogen skin (qPCR/ΔΔCt-method: n = 4; *P<0.05).

Several signaling pathways have been identified which play an important role in processes of folliculogenesis and structural polarization within epithelium, including HF’s. One mechanism pivotal to epithelial organization and differentiation is the Wingless integration (Wnt)-signaling pathway [2], [28], [29], [30], [31], [32], [33]. We first screened for the expression of a series of components of Wnt-signaling and further evaluated Wnt5a/10b ligand, frizzled (Fzd) 5 receptor and Cateninβ transcriptional regulator. We saw that in WT skin Cateninβ levels typically increased with anagen (8.64 fold±3.96 SD) but in the Nu/Nu phenotype they appeared to be uniformly elevated above WT telogen levels (3.92 fold±1.58 SD). Among Wnt-ligands and receptors, Wnt5a and 10b as well as Fzd 1, 3, 4 & 5 showed robust expression levels in both WT & Nu/Nu. However, whereas Wnt5a (12.39 fold±1.07 SD) and mostly Fzd5 (1.85 fold±0.26SD) were upregulated in Nu/Nu over WT telogen, Wnt10b was suppressed (0.26 fold±0.21 SD) (Fig. 5A).

### Epithelium also shows a Heightened Responsiveness to Hedgehog-signaling in the Nu/Nu Phenotype

The sonic hedgehog homolog pathway (Shh)-signaling pathway potentially plays a role in HF stem cell regulation, including the induction of anagen [34], [35], [36], [37], [38]. It consists of Shh ligand, the responsive Patched (Ptch)1 receptor molecule and the interconnected Smoothened (Smo) membrane protein that regulates the transcription factors (TF’s) Gli 1-3. We saw that Shh expression in the hairless-Nu/Nu condition resembles WT telogen and is increased in the lanugo-Nu/Nu condition (11.21 fold±1.4 SD), similar to WT anagen (7.89 fold±0.72 SD). Although Shh transcriptional levels in hairless-Nu/Nu skin appeared to be similar to WT, other pathway components were significantly up-regulated, most distinctly Ptch1 (∼10 fold) and Gli2 (∼20 fold) (Fig. 5B).

### Comparison of in vitro Cultured WT vs. Nu/Nu Derived Epithelial Primaries

**Figure 6:**
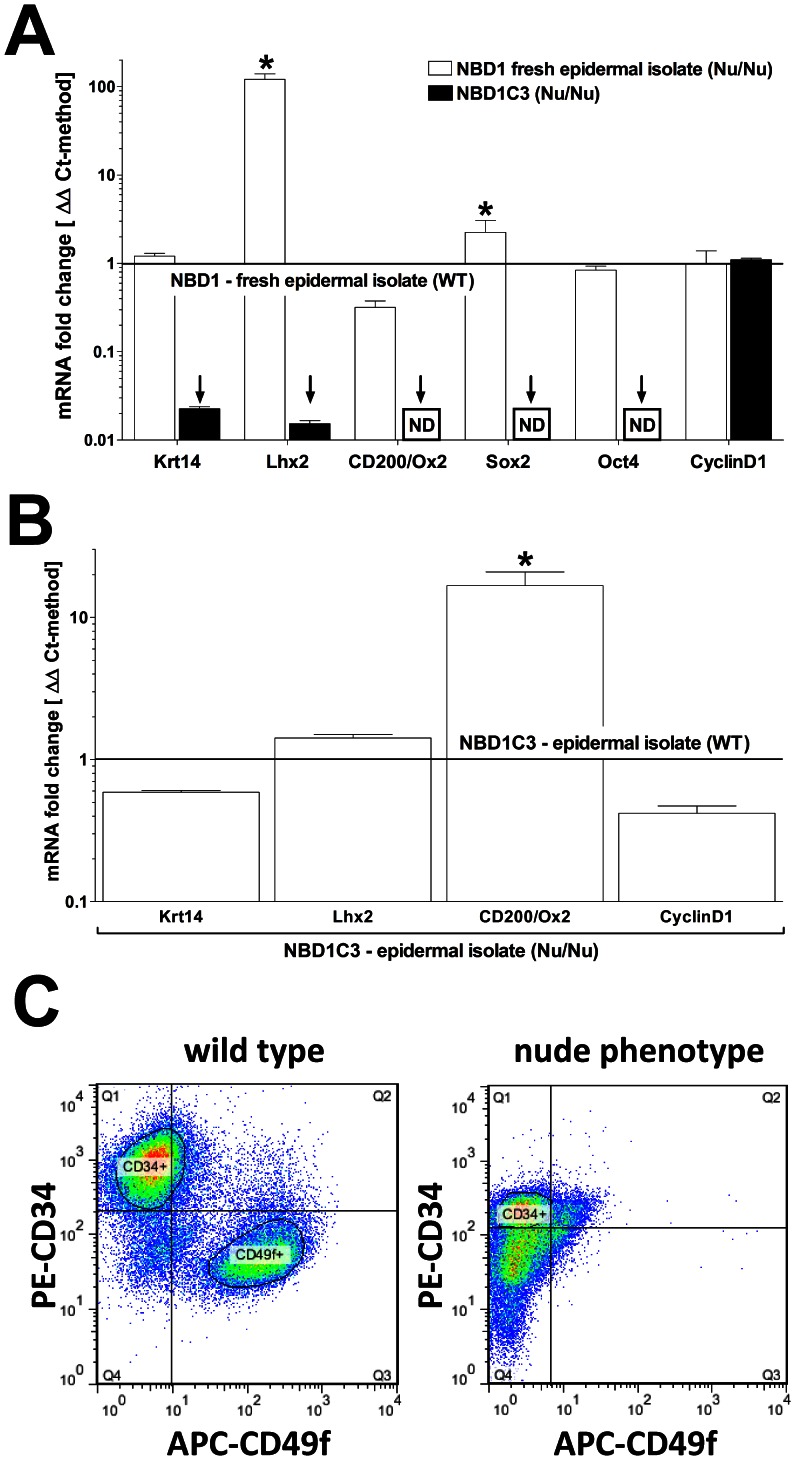
**Aspects of the Foxn1^−/−^ phenotype in epithelial isolates and in vitro.** (A) Newborn epithelial isolates on day one post birth (NBD1) of Foxn1^−/−^ background show strong upregulation of Lhx2 which is not maintained following three days of in vitro culture along with other marker genes of progeny (ND = non-detectable). (B) However, CD200 levels remain higher in cultured Foxn1^−/−^ isolates compared to WT (qPCR/ΔΔCt-method: n = 4; *P<0.05, ↓P<0.05). (C) Flow cytometry on day 5 of in vitro cultures reveals a shift from a dominant CD49f++ subpopulation in vivo in favor of a CD34+/CD49f+ positive epithelial subpopulation in vivo which is more pronounced in Nu/Nu isolates vs. WT.

We also evaluated how Foxn1 loss-of-function will affect keratinocytic lineage differentiation in vitro. For this, primary cells were isolated from newborn (NBD1) skin of both WT and Nu/Nu mice. Total isolates were cultured on collagen I/laminin-coated dishes without feeder cells as previously described [14], [15], [16]. Comparative evaluation of cultures was performed on day 3 (NBD1C3) where single colony forming units were still distinguishable using gene expression analysis and flow cytometry. We first performed comparative gene expression analysis of fresh (NBD1) and cultured (NBD1C3) WT vs. Nu/Nu skin isolates (Fig. 6). Compared to what we saw with adult whole skin (Fig. 3C), fresh epithelial isolates showed an even more pronounced upregulation of Lhx2 expression in Nu/Nu-NBD1 vs. WT-NBD1 by over ∼100 fold along with stabilized CyclinD1 levels. Also, suppression of CD200 mRNA as well as a reduced CD200+ population in Nu/Nu isolates reflected conditions in adult skin. In contrast, we saw elevated Sox2 (∼2 fold) and leveled Oct4 expression in NBD1 isolates opposed to a strongly suppressed expression of both markers in adult, Nu/Nu whole skin. Culture conditions led to a strong downregulation of Lhx2, CD200, CD200R and non-detectable Sox2 and Oct4 levels in both Nu/Nu- and WT-NBD1C3 keratinocytes. However, comparison between cultured isolates with each other revealed that the Foxn1 loss-of-function correlated with significantly higher expression levels of CD200 (∼10 fold) compared to WT-NBD1C3. In contrast with the observed downregulation of marker genes for keratinocytic progeny, Krt14 and CyclinD1 levels remained stable or slightly elevated in cultured compared to fresh isolates. We continued surface marker profiling of cultured isolates using flow cytometry. We observed a virtual absence of an Oct3/4 or CD200 positive population in both cultured Nu/Nu- and WT-NBD1C3 keratinocytes. However, cultured cells showed a distinct separation into a CD34+/CD49f- (49.0%±8.9 SD) and CD49f+/CD34− (26.4%±3.7 SD) population in WT. Conversely, in Nu/Nu-NBD1C3 isolates, we saw a reduced CD34+/CD49f- (28.3%±5.1 SD) and a near absence of a defined CD49f+ population (Fig. 6). Thus, Foxn1 loss-of-function appears to strongly inhibit differentiation of epithelial progenitors into a CD49f+ phenotype which may be compensated for *in vivo* but not *in vitro* by upregulation of Lhx2.

## Discussion

**Figure 7:**
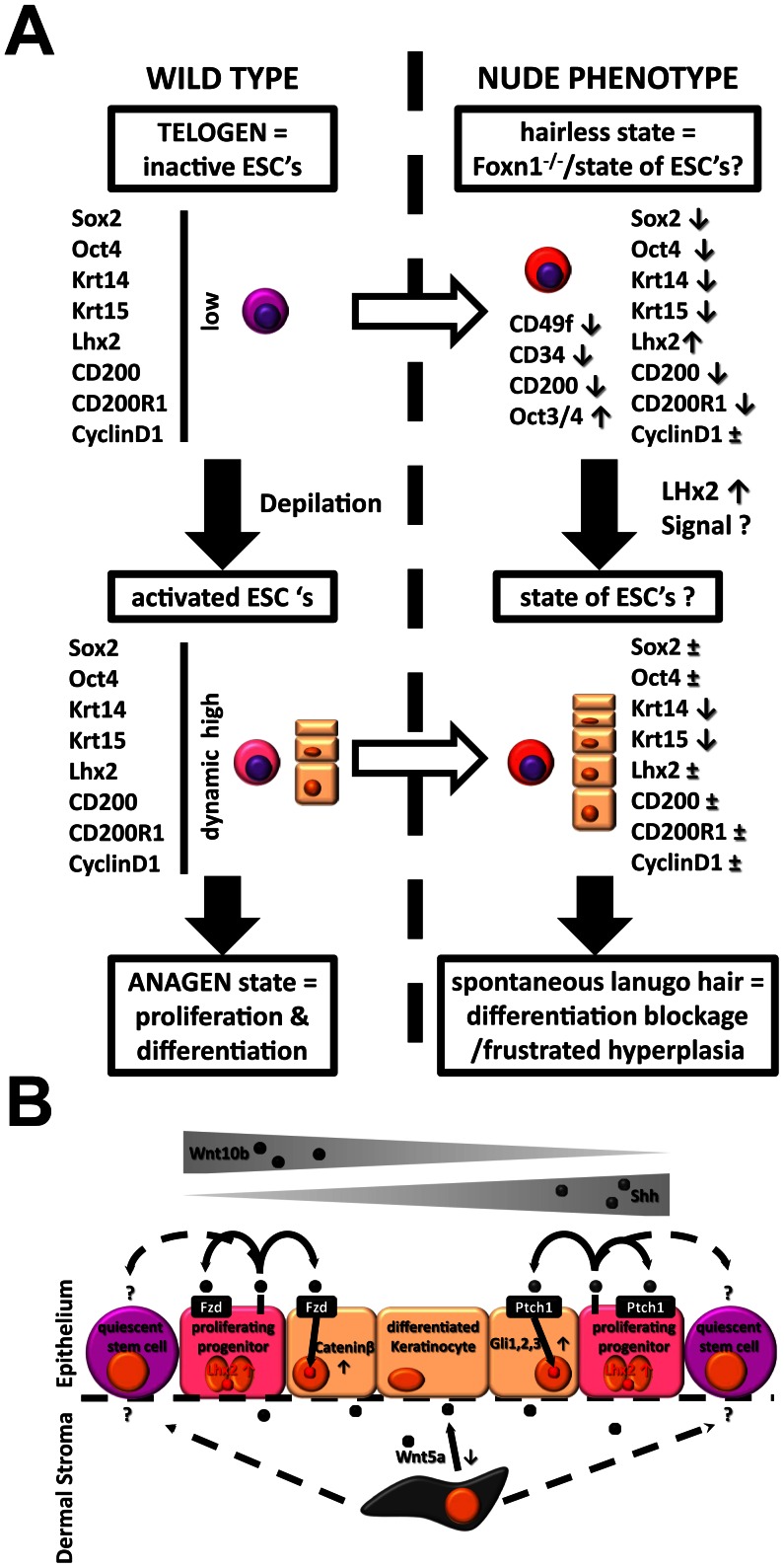
**Schematic of the effects of Foxn1 loss-of-function on keratinocytic lineage differentiation in nude mice.** (A) A severe disturbance in keratin synthesis and terminal differentiation in the Foxn1^−/−^ phenotype also affects upstream lineage progenitors with strong upregulation of Lhx2 as a key feature. (B) Altered Wnt- and Shh-signaling in the Foxn1^−/−^ phenotype.

The hairless appearance of Nu/Nu mice is widely considered a downstream effect resulting from the severe impairment of Foxn1-dependent keratin synthesis. Poorly understood so far however were upstream effects of non-functional Foxn1 expression on keratinocytic lineage differentiation. By comparison to defined telogen and anagen states of a normal hair cycle in WT mice, we were able to confirm an additional disturbance in the regulation of epithelial progeny (summarized Fig. 7A). Most notably, we found Lhx2 [39], [40], a putative regulator of epithelial ‘stem cellness’, constitutively upregulated both compared to the telogen and anagen conditions in WT. However, dynamic changes in Lhx2 expression with spontaneous, rudimentary lanugo hair formation occurred, suggesting a potentially intact hair cycle mechanism in Nu/Nu mice.

Lhx2 null mutations are lethal in an embryonic stage and appear to be crucial during brain development. In embryonic skin, Keratin14 (Krt14) +, α-integrin (CD49f)+and P-cadherin (PCAD) – epidermal progenitor cells in HF placodes showed high Lhx2 expression levels compared to a more differentiated PCAD+cell population in the interfollicular epidermis [39]. In adult WT mice, Lhx2 expression is predominately localized within the bulge region of HF’s. Knockout experiments positioned Lhx2 downstream of Cateninβ and SHH signaling. Also,hair cycle periods are significantly shortened along with a strongly reduced CD34 positive epithelial progenitor cell population. A more detailed, hair cycle dependent profiling concluded that Lhx2 expression peaks during early stages of anagen and is at least briefly silenced during telogen. In transgenic animals it was confirmed that Lhx2 is essential for maintaining anagen hair cycle progression [41]. In conclusion, Lhx2 appears to be an imminent regulator of stem cell differentiation in HF’s. Our own findings of Lhx2 upregulation in Nu/Nu mice argue in favor of a permanent pro-anagen activation of HF’s with a significant impact on mechanisms of stem cell regulation within the bulge.

In this context, we show that in WT mice the induction of a new hair cycle via depilation is characterized by a brief but distinct increase in the expression of embryonic stem cell markers Sox2 and Oct4 during an early phase of anagen. Although HF’s of Nu/Nu mice are able to cycle between the anagen-catagen-telogen states [10], we persistently saw both markers suppressed in Nu/Nu mice. As expected, additional markers of progeny such as Krt14 and 15, which are at least partly under the control of Foxn1, were downregulated at all times. However, the assumed bulge cell markers CD200/Ox2 [24] and CD200-R1 [42] were also strongly suppressed but at least were able to reach WT telogen expression levels under the lanugo-Nu/Nu condition (spontaneous growth of lanugo-type hair). In addition, wound healing studies in heterozygotous Lhx2+/− mice, −/− being lethal, showed a significant downregulation of stem cell associated markers such as Sox9, Tcf4 and Lgr5 [43]. Thus, a one-sided Lhx2 upregulation in Nu/Nu mice suggests a blockage at an early stage of progenitor cell differentiation way upstream of Foxn1 transcriptional control.

Although Lhx2 expression appears to be mandatory regarding the induction of hair growth from a quiescent telogen state, so far no specific epithelial subpopulation has been linked to it. In wild type, a CD49f expressing progenitor typically found in the basal layer of the epidermis [21], [22] is most predominant, followed by CD34 expressing progenitors that are most abundant at the root sheet level of hair follicles below the bulge region [23], [44], [45], [46], [47] (Fig. 1). In addition, a population highly (++) positive for CD49f can typically be distinguished from a population with intermediate (+) positivity. Also, high positivity appears to normally exclude concomitant CD34 or CD200 expression. However, our flow cytometry data confirms that a ‘grey zone’ of CD49f/CD34 double positive, probably less committed cells exists. Our evaluation of fresh newborn epithelial isolates revealed a significant reduction of distinct CD49f or CD34 positive populations in the Nu/Nu phenotype along with the appearance of a novel CD49f++/CD34+ population not seen in WT. This suggests that Foxn1 loss-of-function inhibits the commitment of a common progenitor in turn causing upregulation of Lhx2 perhaps in order to ‘force’ further differentiation. In addition, we also saw a significant reduction of a CD200+ population along with an expansion of Oct3/4 positive cells in dermal, but not epidermal fractions of Nu/Nu isolates. Considering that Lhx2 was also strongly upregulated in fresh isolates of Nu/Nu newborn mice this again implies Lhx2 as a key component of bulge stem cell regulation. However, our observation in newborn skin does not appear to hold true for the adult Nu/Nu phenotype, were Sox2 and Oct4 levels are suppressed. This could indicate that within immature, nascent hair follicles of newborn skin a hierarchy within a bulge progenitor cell population has yet to be established from a more uniform, least differentiated stem cell population.

To further characterize the uniqueness of the Foxn1 deletion-mutation in respect to progenitor cell regulation in epithelium, we further evaluated signaling pathways which are well known regulators of morphogenesis (summarized in Fig. 7B). Wnt-signaling is required for embryonic and de novo formation of HF’s in adult skin e.g. in the context of wound healing [2] and in the thymus Wnt-signaling has been shown to control the transcription of Foxn1 [9]. Overexpression of the canonical intracellular Wnt-signaling transcriptional modulator Cateninβ is sufficient to induce anagen. In stem cells, Cateninβ negatively regulates Nanog [48], a gene associated with pluripotency whereas overexpression of Oct4 can induce Cateninβ signaling [49]. Most notably our own data showed a >10-fold increase of Wnt5a in hairless-Nu/Nu skin over WT telogen and less so of Cateninβ and Fzd5. Conversely, Wnt10b, an important inducer of differentiation within surface epithelium and inner root sheet (IRS) cells of HF’s during anagen [30] was suppressed. In addition, CyclinD1 expression in both Nu/Nu and WT telogen was at identical baseline levels. Thus the observed modulation in Wnt-signaling is likely to reflect another abnormality in bulge stem cell regulation. Our findings suggest that Wnt10b and therefore Cateninβ-dependent gene expression is negatively regulated by Lhx2 within bulge cells. A modulation of Wnt5a expression however has to be seen in a context of interactions between bulge stem cells with neighboring mesenchym. Immunolocalization found Wnt5a strongly expressed within the mesenchymal-derived dermal papilla of anagen HF’s but also by matrix and IRS cells and expression regressed to the bulge area with the beginning of catagen [50]. A non-canonical, Wnt5a-mediated rise in intracellular calcium acts antagonistic to canonical, Cateninβ-mediated signaling. Thus, our findings indicate that Lhx2 induces Wnt5a secretion within epithelium and/or indirectly in mesenchymal lineage cells adjacent to HF’s. The latter could be the first demonstration of how epithelial progenitors prepare their non-epithelial environment for an impending anagen, proliferative expansion.

Closely related to Wnt-signaling, sonic hedgehog homolog (Shh) is the ligand of another important gradient-driven signaling pathway that has been shown active during adult anagen HF growth and is an important regulator of cell cycle progression in epithelial progenitors [51]. Loss-of-function studies of both Shh and Gli TF’s showed that the Shh-pathway is crucial for HF formation and cycling but not for epidermal differentiation [52]. In the embryo, Shh secretion is a function of mesoderm (e.g. notochord) with exerts an influence on ectoderm (e.g. neural tube) [53] and can be considered a functional antagonist of the Wnt-pathway [54]. Regarding de novo HF formation and HF cycling, the canonical Wnt-pathway has to be activated before expression of Shh-pathway components can occur [55]. We saw that in Nu/Nu skin Foxn1 loss-of-function did not appear to directly affect Shh expression compared to WT. However, we saw most other components related to Shh-signaling upregulated which possibly reflects an increased responsiveness of epithelial progenitors to mesenchymal Shh secretion under the influence of Lhx2.

The study of progenitor/stem regulation in skin so far has been largely limited to *in vivo* experimental approaches. Current culture methods have been significantly improved in recent years in regards to maintaining a distinct keratinocytic phenotype [56], e.g. with continued expression of keratins or surface markers such as CD49f but they remain poorly suited to study epithelial progeny. So far a higher mitogenic propensity/CFU-properties of CD49f, CD34 and CD200 expressing epithelial primaries is well documented [47], [57], but current culture methods are still lacking in their ability to maintain those properties [26]. We investigated possible implications of Foxn1 loss-of-function under cell culture conditions. As previously described [26], CD200 expression was quickly lost or strongly downregulated from cultured wild type isolates along with several other markers of progeny. However, cultured keratinocytes of wild type origin usually continue to express CD49f and CD34. Surprisingly, cultured Nu/Nu-NBD1C3 isolates diverged from the wild type condition by a strong reduction of a CD49f expressing population whereas a CD34+ population was maintained, indicating a more selective impact of Lhx2 upregulation in the Foxn1^−/−^ phenotype on different branches of epithelial lineage differentiation. Overall, our *in vitro* data indicates that with current cell culture methods, specifically under low calcium conditions with the addition of various growth promoting supplements, primary cells of the keratinocytic lineage are able to maintain a CD34 and CD49f positive epithelial phenotype but otherwise will quickly loose several markers of progeny including Oct4, Sox2, CD200 as well as Lhx2 expression.

In conclusion, our findings show that the Foxn1^−/−^ mutation has a relevant impact on downstream mechanisms of terminal keratinocytic differentiation which result in the hairless phenotype. As evidenced by upstream ‘gateway-keepers’ of differentiation such as Lhx2 it also profoundly affects stem cell niches within the skin. The Nu/Nu phenotype is also likely to offer novel insights in HF morphogenesis and cycling related to interactions between the epithelial and mesenchymal cell lineage with several signaling pathways such as that related to bone morphogenetic proteins (BMP’s) [58], [59], tumor growth factor β (TGF-β) [60] or insulin like growth factor 1 (IGF-1) [61] being worthwhile further investigation.
